# Laser Materials Fabrication and Joining

**DOI:** 10.3390/ma13122800

**Published:** 2020-06-22

**Authors:** Giuseppe Casalino

**Affiliations:** Dip. di Meccanica, Matematica e Management, DMMM-Polytechnic University of Bari, Via Orabona 4, 70125 Bari, Italy; giuseppe.casalino@poliba.it

**Keywords:** laser manufacturing, experiments, numerical simulation, laser material interaction

## Abstract

This laser special issue has brought together academic scientists, researchers and laser manufacturers for a share of their experiences on recent progress in laser science and technology in the fields of laser materials processing for manufacturing. The papers cover advances in laser materials fabrication and joining of emerging materials, their fabrication and application.

Laser-based manufacturing has increased dramatically in many different industries with unprecedented precision, efficiency and variety in materials processing [1]. High flexibility, efficiency, reproducible adjustability of processing parameters, and excellent quality of processed products are the main advantages that are provided by the laser-based technologies, which have opened the door to previously non-existent processes like direct metal deposition, laser sintering and melting, and laser powder welding [2,3,4].

This Special Issue concerns the application of the laser technology to welding advanced materials and dissimilar joints, along with additive fabrication, material trepanning, composite pre-treatment, surface treatment, and laser component fabrication. The aims of the papers of this Special Issue are to raise the scientific and technical attention and interest for laser material processing. In more detail, the presented papers deal with the following subjects.

The hybrid laser-MAG (metal active gas) welding process of twinning-induced plasticity and dual-phase steels with austenitic stainless steel (AISI316) was simulated by means of a thermo-mechanical model, which was developed using the finite element method [5].

The microstructural and mechanical properties of laser–tungsten inert gas hybrid welding of Mg alloy sheets for automobiles were investigated including AZ31 and ME21, AZ31 and AZ31, ME21 and ME21 [6].

The thermal efficiency analysis of the Laser-Assisted Plasma Arc Welding of AISI 304 Stainless was related to the melting efficiency for different sizes of the heat sources [7].

Titanium and Aluminum alloys were welded using the so-called off-set technique. The effects of focus and off-set distance of the laser beam on the weldability of grade 5 titanium to 6061 Aluminum alloy dissimilar butt weld were assessed [8].

The 10-mm thick high-yield-point steel S700MC was tested for disc laser welding for single- and double-sided welding [9].

A concise index of some governing factors with a potential operational use was proposed for Inconel 718 laser powder bed fusion. It was called volumetric energy density [10].

Samples of a WC/Co/Cr composite powder were fabricated and characterized in terms of density, defects, microstructure and hardness [11].

A comprehensive numerical model for the process of laser-assisted deposition of Aluminum alloy 2024 was built to assess the most effective reference shape to feed the simulation as a function of the governing factors in advance [12].

The applicability of laser-assisted synthesis for producing high-density Cu-Al-Ni alloys with shape memory characteristics was studied with the goal of further development towards a method of additive manufacturing of large-sized Cu-based shape memory alloys [13].

Laser surface transformation hardening of AISI 4130 was investigated by a Nd:YAG pulsed laser. Laser focal height, pulse width, scanning speed, and power varied during the experiments [14].

Laser shock processing was utilized to strengthen the cavitation erosion resistance of austenitic stainless-steel laser weldments [15].

A semi-water-immersed laser micro-trepanning process was investigated with alumina ceramics as the target material. The performance was assessed and compared with the direct laser trepanning method [16].

An experimental investigation of the machining performance of the direct and chemical-assisted picosecond laser trepanning of single crystalline silicon was conducted to assess the machining method [17].

Using different laser sources, a composite aerospace prepreg system underwent laser-based bonding pre-treatment with analytical tests and after-bond mechanical testing to determine the bonding ability of the specimen treatment [18].

The silica is widely applied in the modern laser system. A highly-efficient and low-damaging lapping process model was presented for the optimization of the finishing operation of silica [19].

## References

[1] Rashkovets M., Mazzarisi M., Nikulina A.A., Casalino G. (2020). Analysis of laser direct stainless steel powder deposition on Ti6Al4V substrate. Mater. Lett..

[2] Campanelli S.L., Angelastro A., Signorile C.G., Casalino G. (2017). Investigation on direct laser powder deposition of 18 Ni (300) marage steel using mathematical model and experimental characterization. Int. J. Adv. Manuf. Technol..

[3] Casalino G., Campanelli S.L., Contuzzi N., Ludovico A.D. (2015). Experimental investigation and statistical optimisation of the selective laser melting process of a maraging steel. Opt. Laser Technol..

[4] Errico V., Campanelli S.L., Angelastro A., Mazzarisi M., Casalino G. (2020). On the feasibility of AISI 304 stainless steel laser welding with metal powder. J. Manuf. Process..

[5] Perulli P., Dassisti M., Casalino G. (2020). Thermo-Mechanical Simulation of Hybrid Welding of DP/AISI 316 and TWIP/AISI 316 Dissimilar Weld. Materials.

[6] Li T., Song G., Zhang Z., Liu L. (2019). Mechanical Properties and Microstructures of Laser–TIG Welded ME21 Rare Earth Mg Alloy. Materials.

[7] Hipp D., Mahrle A., Beyer E., Jäckel S., Hertel M., Füssel U. (2019). Thermal Efficiency Analysis for Laser-Assisted Plasma Arc Welding of AISI 304 Stainless Steel. Materials.

[8] Casalino G., D’Ostuni S., Guglielmi P., Leo P., Palumbo G., Piccininni A. (2018). Off-Set and Focus Effects on Grade 5 Titanium to 6061 Aluminum Alloy Fiber Laser Weld. Materials.

[9] Górka J. (2018). Assessment of the Weldability of T-Welded Joints in 10 mm Thick TMCP Steel Using Laser Beam. Materials.

[10] Caiazzo F., Alfieri V., Casalino G. (2020). On the Relevance of Volumetric Energy Density in the Investigation of Inconel 718 Laser Powder Bed Fusion. Materials.

[11] Campanelli S.L., Contuzzi N., Posa P., Angelastro A. (2019). Printability and Microstructure of Selective Laser Melting of WC/Co/Cr Powder. Materials.

[12] Caiazzo F., Alfieri V. (2019). Simulation of Laser-assisted Directed Energy Deposition of Aluminum Powder: Prediction of Geometry and Temperature Evolution. Materials.

[13] Niedbalski S., Durán A., Walczak M., Ramos-Grez J.A. (2019). Laser-Assisted Synthesis of Cu-Al-Ni Shape Memory Alloys: Effect of Inert Gas Pressure and Ni Content. Materials.

[14] Casalino G., Moradi M., Moghadam M.K., Khorram A., Perulli P. (2019). Experimental and Numerical Study of AISI 4130 Steel Surface Hardening by Pulsed Nd:YAG Laser. Materials.

[15] Zhang L., Liu Y.-H., Luo K.-Y., Zhang Y.-K., Zhao Y., Huang J.-Y., Wu X.-D., Zhou C. (2018). Tensile Property of ANSI 304 Stainless Steel Weldments Subjected to Cavitation Erosion Based on Treatment of Laser Shock Processing. Materials.

[16] Ma Q., Zhu H., Zhang Z., Xu K., Dai X., Zhu S., Wang A. (2019). An Investigation into Picosecond Laser Micro-Trepanning of Alumina Ceramics Employing a Semi-Water-Immersed Scheme. Materials.

[17] Zhu H., Zhang Z., Xu K., Xu J., Zhu S., Wang A., Qi H. (2019). Performance Evaluation and Comparison between Direct and Chemical-Assisted Picosecond Laser Micro-Trepanning of Single Crystalline Silicon. Materials.

[18] Blass D., Nyga S., Jungbluth B., Hoffmann H.D., Dilger K. (2018). Composite Bonding Pre-Treatment with Laser Radiation of 3 µm Wavelength: Comparison with Conventional Laser Sources. Materials.

[19] Song C., Shi F., Zhang W., Lin Z., Lin Y. (2020). High-Efficiency and Low-Damage Lapping Process Optimization. Materials.

